# Hospital based surveillance of congenital rubella syndrome cases in the pre-vaccine era in Amhara Regional State, Ethiopia: A base line information for the country

**DOI:** 10.1371/journal.pone.0207095

**Published:** 2018-11-12

**Authors:** Yitayih Wondimeneh, Moges Tiruneh, Getachew Ferede, Kassahun Denekew, Fisseha Admassu, Belay Tessema

**Affiliations:** 1 Department of Medical Microbiology, School of Biomedical and Laboratory Sciences, College of Medicine and Health Sciences, University of Gondar, Gondar, Ethiopia; 2 Department of Pediatrics, School of Medicine, College of Medicine and Health Sciences, University of Gondar, Gondar, Ethiopia; 3 Department of Ophthalmology, School of Medicine, College of Medicine and Health Sciences, University of Gondar, Gondar, Ethiopia; Universita degli Studi di Bologna, ITALY

## Abstract

**Background:**

Rubella virus infection in early pregnancy lead to serious multi-organ birth defects known as congenital rubella syndrome (CRS). The incidence of CRS varies in different populations and the highest burden is found in developing countries in which rubella vaccination is not included in their national immunization programs. In Ethiopia, there is scarcity of data about congenital rubella syndrome. Therefore, the aim of this study was to determine the burden of CRS-related birth defects and its incidence in the pre-vaccine era in Amhara Regional State, Ethiopia.

**Materials and methods:**

A cross sectional study was conducted in Dessie, Felege-Hiwot and University of Gondar Referral Hospitals, from December 2015 to August 2017. After getting informed assent from each parent/guardian, blood was collected from infants < 1 year of age for laboratory determination of anti-rubella virus antibodies. Their socio-demographic data and clinical information compatible with congenital rubella syndrome were collected using WHO guideline.

**Results:**

During the study period, a total of 50 infants suspected for congenital rubella syndrome were included in the study. All infants suspected for CRS were tested against rubella specific IgM and IgG [for infants ≥ 6 months of age] antibodies using ELISA method. Of these, 9/50 (18%) and 4/14 (28.6%) of them were laboratory confirmed and potential CRS cases, respectively. In the present study, the most common laboratory confirmed defect was ocular manifestations 6 (66.7%) followed by heart related problems 5 (55.6%). In the present study, most of the laboratory confirmed cases (66.7%) were reported among 1–5 months of age infants. In addition, 5 (55.6%) of the infants with laboratory confirmed CRS cases were male and 6 (66.7%) of them were from urban settings. In this study, the incidence of CRS was 0.4 per 1000 live births.

**Conclusion:**

In this study, nearly one fifth of the infants had laboratory confirmed congenital rubella syndrome and most of them had multiple rubella associated congenital defects at a time. Most of these congenital anomalies were reported among infants ≥ 1 month of age. Based on our result, the incidence of the CRS was line with the global incidence of the CRS in the pre-vaccine era. Therefore, establishing strong rubella/CRS surveillance system as well as introducing the rubella containing vaccine in the national immunization program might be important to reduce the burden of rubella and CRS in the country.

## Introduction

Rubella virus (RV) infection in the early stages of pregnancy can lead to serious birth defects known as congenital rubella syndrome (CRS) [1]. As CRS is a multi-organ disease that can involve almost every organ of the body [2, 3], infants with CRS frequently exhibit both intrauterine and postnatal growth retardation [3]. In addition, children exposed to the virus prenatally may be born with rubella associated malformations such as hearing loss, blindness, cardiac defects, acute meningoencephalitis and pan encephalitis. Furthermore, the risk of intellectual disability and behavioral problems can be also increased in children with CRS [4, 5].

In 1996, around 119 (range 72,000–169,000) CRS cases were estimated globally as compared to 105,000 (ranged: 54,000–158,000) in 2010 [6]. In 1996, an estimated 22, 000 babies were born with CRS in Africa, along with about 46, 000 CRS in South-East Asia, and close to 13 000 in the Western Pacific [7]. In 2010, 39,000 (ranged: 18,000–80,000) and 49,000 (ranged: 11,000–97,000) CRS cases has been predicted in Africa and South-East Asia, respectively [6]. However, only few countries have introduced rubella vaccination in their national immunization programs [8] and the lack of vaccination programme in children might contribute to the high incidence of CRS cases [9]. Therefore, the current burden of CRS cases in some of these regions is thought to be almost similar to previously estimated [10]. Furthermore, due to the lack of strong surveillance system, the CRS cases are rarely reported in the developing countries and the extent of the problem remains unknown [11]. In addition, since many countries in Africa, Eastern Mediterranean and Southeast Asia didn’t include the rubella vaccination in their national immunization program [12], these regions are known to have the highest burden of rubella and CRS [13, 14]. The highest risk of CRS has been reported in countries with high susceptibility to rubella infection among women of childbearing age [15].

As the CRS cases have diverse form of clinical patterns and treatment modalities [16], early recognition of CRS cases is crucial for physicians to help the patients. However, suspected CRS case identification can be challenging since clinical symptoms such as sensorineural hearing loss are often not clinically apparent immediately after births [16, 17]. Children born asymptomatic might develop these abnormalities lately in life. Individuals with CRS have an increased risk of developing endocrinopathies such as diabetes mellitus and thyroid problems [18–20]. In addition, children with CRS may have also behavioral disorders and panencephalitis later in their life [20]. Therefore, patients with CRS would require long-term follow-up and lifelong care to detect progression and manage complications [21, 22]. Due to the complex nature of the CRS cases, a multidisciplinary team approach like medical, surgical, educational and rehabilitative managements is required. For example, infants with CRS might need special educational support and rehabilitation during their adulthoods [23]. Congenital heart defects and cataracts can be corrected by direct surgery [24]. In addition, the management of ocular manifestations like congenital glaucoma, diminished vision, nystagmus and congenital cataract might need also counseling, regular monitoring and provision of low vision devices as needed [25]. Therefore, strong and regular CRS surveillance system is important to follow-up the CRS cases in the community and to refer the suspected cases in to specialized health professionals to help the patient and minimize further CRS associated problems. It is also important to show the magnitude of the problem in a given country [26].

In Ethiopia, there is scarcity of data about CRS and as to the best of our knowledge, there is only one published CRS case report in the Southwest part of the country [27]. Furthermore, like in many African countries [28], CRS surveillance system has not yet been established [29] and the extent of the problem is not known in the country. Therefore, the aim of this study was to determine the burden of CRS-related birth defects and its incidence in the pre-vaccine era in Amhara Regional State, Ethiopia. This report may be used as a base line data for health decision makers and stake holder organizations for remedial action in the country.

## Materials and methods

### Study design, study area and period

A cross sectional study was conducted in three Amhara Regional State Referral Hospitals, namely Felege-Hiwot, Dessie and University of Gondar Referral Hospitals, from December 2015 to August 2017. Felege-Hiwot referral hospital is found in Bahir-Dar, which is located in the Northwest part of Ethiopia and it is the capital city of the Amhara Regional State. Dessie and University of Gondar referral hospitals are 474km and 175km far from Bahir-Dar, respectively. The respective referral hospitals have specializations in internal medicine, pediatrics, gynecology, surgery, ophthalmology and other health related specializations and they also act as teaching hospitals or clinical attachment sites for different health professionals. Of the seven referral hospitals in the region, lottery method was used to select the three hospitals.

### Study participants

Our study participants were all < 1year infants who had been suspected for CRS and visited pediatric outpatient departments (PEDI- OPD), pediatric wards (PEDI-WARD), neonatology wards (NICU) and the ophthalmology clinics of the respective referral hospitals during the study period. Infants suspected for CRS were selected based on WHO guideline [30] (Table 1).

**Table 1:** CRS cases classified to the WHO case definitions.

CRS case category	Case descriptions
Suspected CRS cases	A health worker should suspect CRS when an infant less than one year of aged presents with heart disease and/or suspicion of deafness and/or one or more of the following eye signs: white pupil (cataract), diminished vision, pendular movement of the eyes (nystagmus), squint eyed, smaller eye ball (microphthalmus), or larger eye ball (congenital glaucoma). A health worker should also suspect CRS when an infant’s mother has a history of suspected or laboratory confirmed rubella during pregnancy.
Clinically confirmed CRS cases	A child in whom a physician detects at least two of the complications listed in (A) below or one in (A) and one in (B). **Group A:** cataract(s), congenital glaucoma, congenital heart disease, sensorineural hearing impairment and pigmentary retinopathy. **Group B:** purpura, splenomegaly, microcephaly, developmental delay, meningocephalitis, radiolucent bone disease, and jaundice with onset within 24hours of birth.
Laboratory confirmed CRS cases	A child with clinically confirmed CRS who has a positive blood test for rubella specific IgM antibody
Congenital rubella infection only (CRI)	An infant born from rubella suspected or confirmed mother and who does not have clinical signs of CRS but who have a positive rubella specific IgM test without clinical signs of CRS.
Discarded CRS cases	A child suspected for CRS but have rubella specific IgM and IgG (for infants ≥ 6 months of age) negative test results.

**Key**: The congenital rubella complications listed in groups A and B can be also classified as major and minor signs or symptoms, respectively [17].

### Inclusion and exclusion criteria

The children who fulfilled the cases classified to the WHO case definitions and gave the required amount of blood sample for laboratory analysis were included in the study. But we have excluded the children whose parents/guardians refused to give assent. In addition, children who visited the respective referral hospitals for the same congenital anomalies in the second time during the study period were excluded from the study.

### Data collection instruments

After getting the informed assent from each child’s parent/guardian, socio-demographic data, clinical information compatible with CRS and other relevant information were collected by pediatricians, ophthalmologists, ophthalmic nurses and public health officers (HO).

### Blood collection, handling and transportation

Using plain tube, 1ml [Scalp vein set 23G x3/4”] of venous blood was collected aseptically from each study participant by well-trained medical laboratory professionals and nurses for the laboratory analysis of rubella virus infection. Then the blood was immediately transported in to the respective referral hospital laboratories for serum separation and storage. Blood was allowed to clot for 1 hour at room temperature, centrifuged at 3500 rpm for 5 minutes, and then serum was separated and collected in aliquots in sterile storage vials and stored at-20°C until it is transported into the School of Biomedical and Laboratory Sciences, University of Gondar, using a cold box with frozen ice packs to maintain a condition of about 4–8°C and then the serum was stored at -70°C until laboratory analysis.

### Laboratory analysis and result interpretations

All the serum samples were tested for rubella specific IgM antibody. We have also determined rubella specific IgG antibody for infants ≥ 6 months by using Enzyme Linked Immuno Sorbent Assay (ELIA) [Linear Chemicals, S.L, Spain] method as per manufacturer instructions [31] in the School of Biomedical and Laboratory Sciences, University of Gondar. The results were read by a micro well reader at 450nm compared in a parallel manner with calibrator and controls. For rubella specific IgM, the qualitative result was interpreted as positive if the rubella IgM index was > 1.1, negative when the index was < 0.9 and equivocal when the index was ≥ 0.9 and ≤1.1. For the quantitative determination of rubella specific IgG antibody, the IgG result was expressed in international units per milliliter (IU/ml). According to the manufacturer’s instruction, the IgG result was interpreted as positive when the IgG index-value was >10 IU/ml, equivocal when the index-value was 5–10 IU/ml and negative when the index-value was <5 IU/ml.

### Quality assurance mechanisms

For rubella virus serological testing, the test kits (IgM and IgG ELISA Kits) have their own positive and negative quality control materials that can be run with patient samples and all the test procedures were done strictly following the manufacturer’s instructions. In addition, the standard operational procedures were strictly followed to avoid preanalytical, analytical and post analytical errors during laboratory analysis. In addition, the inclusion and exclusion criteria of the CRS cases were given for data collectors before starting the study. Training was given for data collectors and they were supervised by the research team regularly.

### Data analysis procedure

Data were checked for completeness, cleaned manually, entered and analyzed using SPSS version 20 statistical package. Data were summarized using frequency tables and graphs. For continuous variable, we used median and interquartile range (IQR).

### Ethical consideration

The study was conducted after obtaining institutional ethical clearance from University of Gondar Ethical Review Board (UOG-IRB). Letter of agreement and cooperation was obtained from each referral hospital clinical director/chief executive officer (CEO). The purpose and importance of the study was explained for the study participants’ parents prior to their participation. The information obtained from each study participants was kept confidentially in the file cabinet and was not disclosed to the third party. The parents have right to withdraw their child from the study at any time without affecting the service intended to get from the health institutes. Informed written assent was also obtained from each child parents by the data collector as per the National Research Ethics Review Guideline [32].

## Results

### Sociodemographic characteristics of the infants with CRS cases

During the study period, a total of 4094 infants less than one year with different health related problems were visited the respective referral hospitals. However, based on the cases classified to the WHO case definition, 50 infants suspected for CRS were included in the study. Of these, 17 (34%) of the CRS suspected cases were from Felege-Hiwot, 23 (46%) of them were from University of Gondar and 10 (20%) of the case were from Dessie Referral Hospitals. According to the present study, 3 (6%) and 2 (4%) of the infants suspected for CRS were born at home and private health facilities, respectively. However, 45 (90%) of them were born in the respective referral hospitals. Of the total CRS cases, 36 (72%) of them were in the age group of less than six months and the remaining 14 (28%) of them were ≥ 6 months of age. The median age of the study participants was 2.5 months (IQR: 0.27–9.0). Of the total infants with CRS cases, 29 (58%) were males, 36 (72%) of them were from urban settings (Table 2).

**Table 2:** Sociodemographic characteristics of the infants suspected for CRS in Amhara Regional State Referral Hospitals, Northwest Ethiopia, December 2015-August 2017.

Sociodemographic data	Frequency	Percentage
Age group		
<6 months	36	72
≥6 months	14	28
**Sex**		
Male	29	58
Female	21	42
**Residence**		
Urban	36	72
Rural	14	28

### Clinical manifestations of the infants suspected for CRS

**Fig 1:**
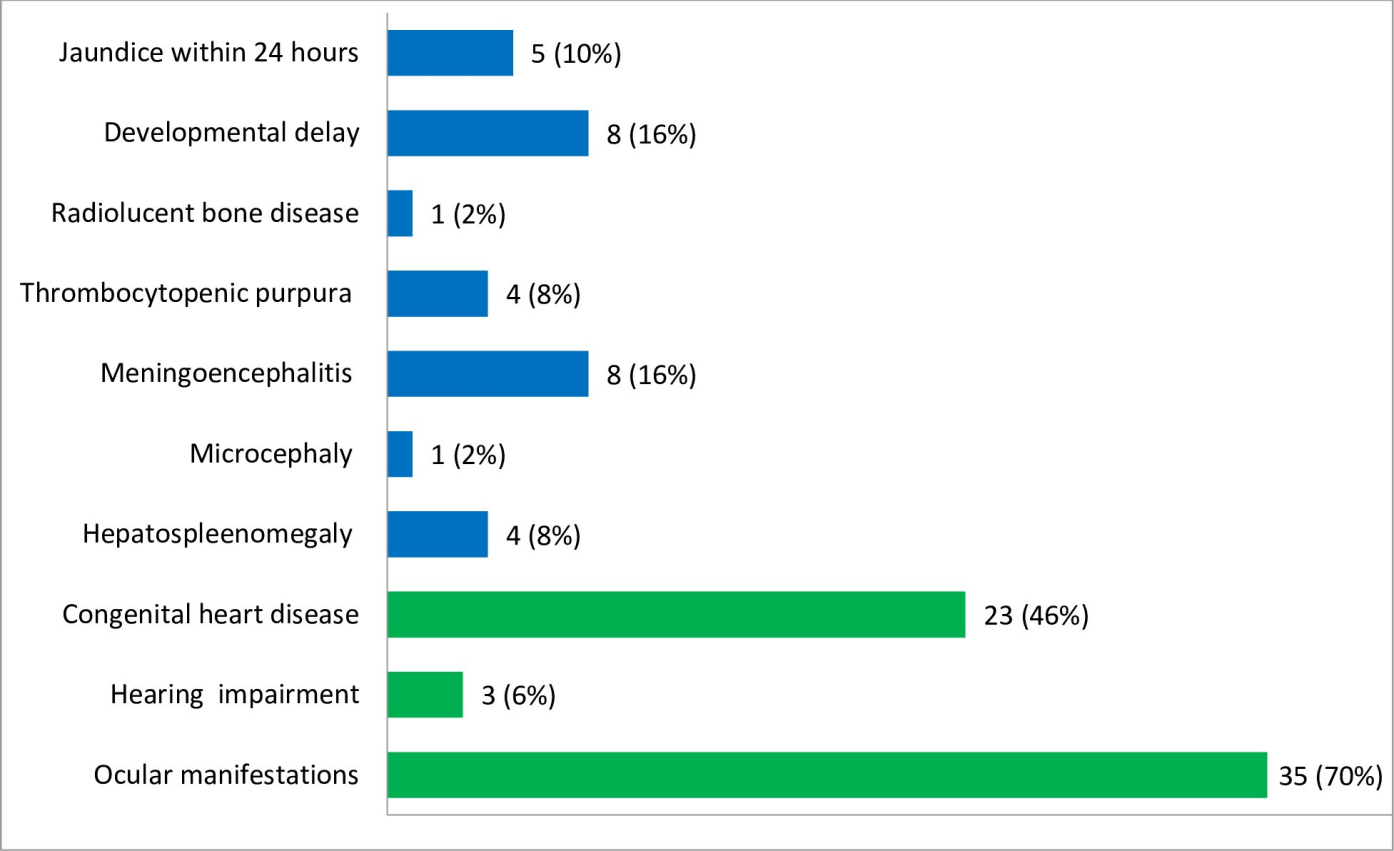
The frequency of different clinical manifestations among infants suspected for CRS in Amhara Regional State Referral Hospitals, Northwest Ethiopia, December 2015-August 2017. The green and blue colors in the bar chart indicate the frequency of each major and minor clinical manifestation of CRS suspected cases, respectively.

**Fig 2:**
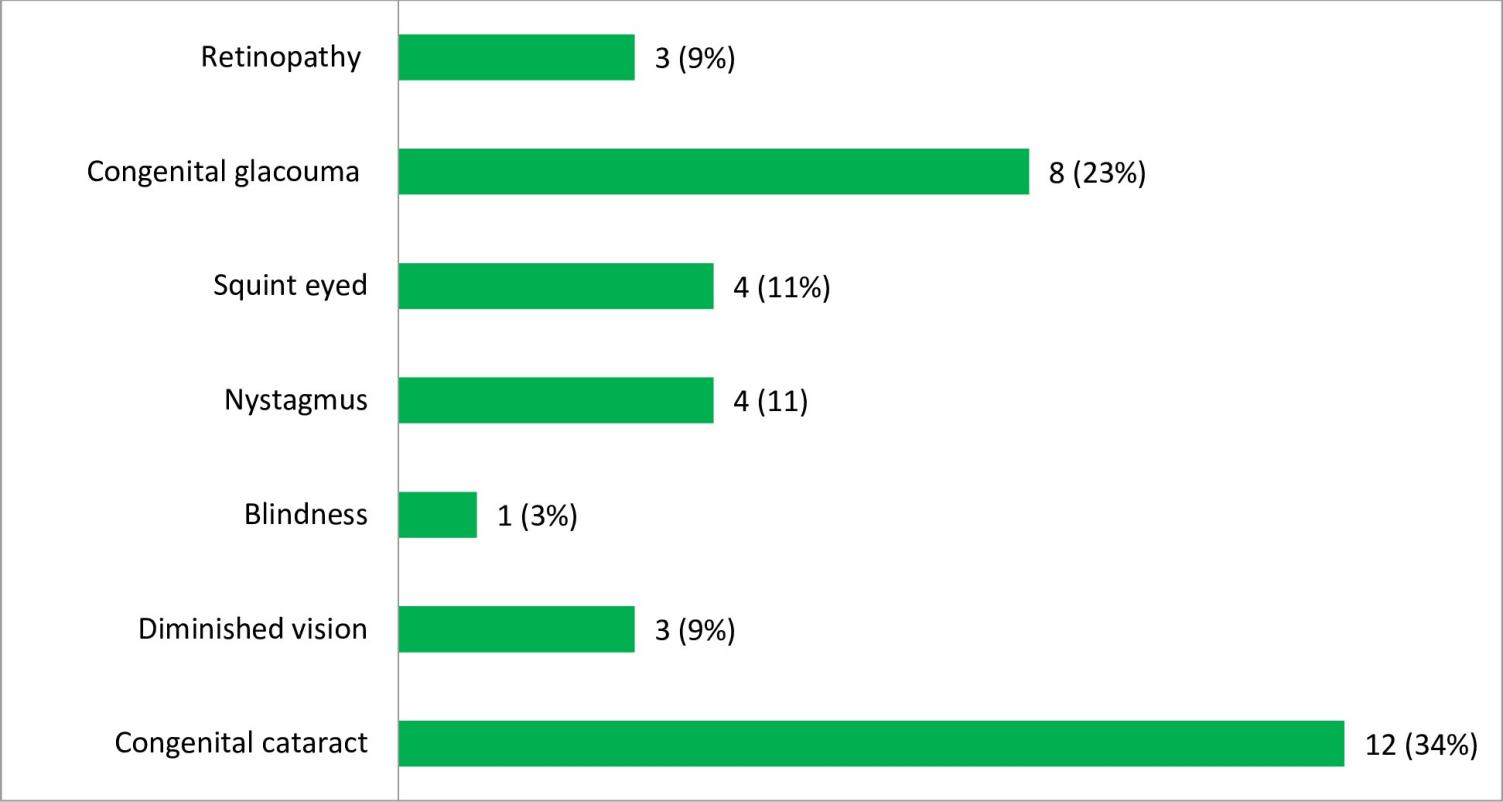
The frequency of different ocular manifestations among infants suspected for CRS in Amhara Regional State Referral Hospitals, Northwest Ethiopia, December 2015-August 2017.

In the present study, the most common major signs of clinical presentation among infants suspected for CRS was ocular manifestations, 35/50 (70%), followed by heart related problems, 23/50 (46%). In addition, 3/50 (6%) of the infants had hearing impairment. Among the minor signs of CRS cases, each of the meningoencephalitis and developmental delay accounts 8/50 (16%) and 5/50 (10%) of them had neonatal jaundice (Fig 1). Among the ocular manifestations, 12/35 (34%) and 8/50 (23%) of the cases were congenital cataract and glaucoma, respectively. In addition, each of the pendular moment of the eyes (nystagmus) and squint eyed accounts 4/35 (11%) of the ocular manifestations. Furthermore, diminished vision and pigmentary retinopathy cases accounted 3/35 (9%) of the ocular manifestations. There was only 1/35 (3%) blinded case (Fig 2).

### Laboratory confirmations of the congenital rubella syndrome cases

**Fig 3:**
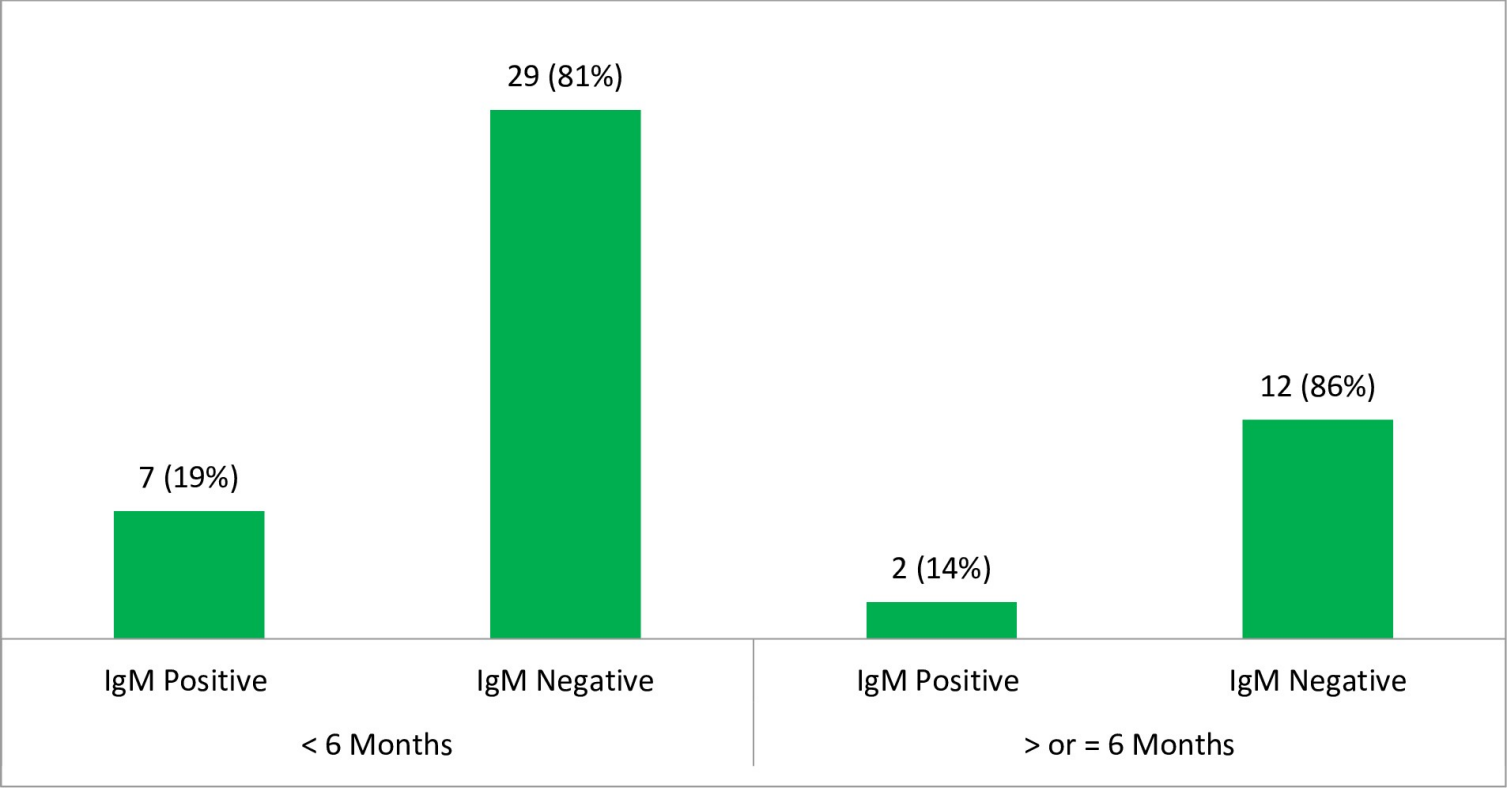
Rubella specific IgM positivity rate in the different age categories of infants suspected for CRS in Amhara Regional State Referral Hospitals, Northwest Ethiopia, December 2015-August 2017.

In the present study, each of the CRS suspected cases were tested against rubella specific IgM antibody. Of the total CRS cases, only 9/50 (18%) of them were positive for rubella specific IgM antibody. According to the cases classified to the WHO case definitions, all the IgM positive cases can be classified as laboratory confirmed CRS cases. When we see the laboratory confirmed CRS cases in relation to the age categories, 7/36 (19%) of the cases in the age group of less than six months were IgM positive. Of the total 14 CRS suspected cases in the age of ≥ 6 months, 2/14 (14%) of them were also IgM positive (Fig 3). In this study, we have also determined the rubella specific IgG antibody for infants ≥ 6 months. Of the total infants in this age category, 4/14 (29%) of them were positive only for rubella specific IgG antibody. These can be considered as potential CRS cases. However, remaining 37/50 (74%) of the CRS suspected cases were negative for both rubella specific IgM and IgG antibodies and can be referred to as none CRS or discarded cases.

**Fig 4:**
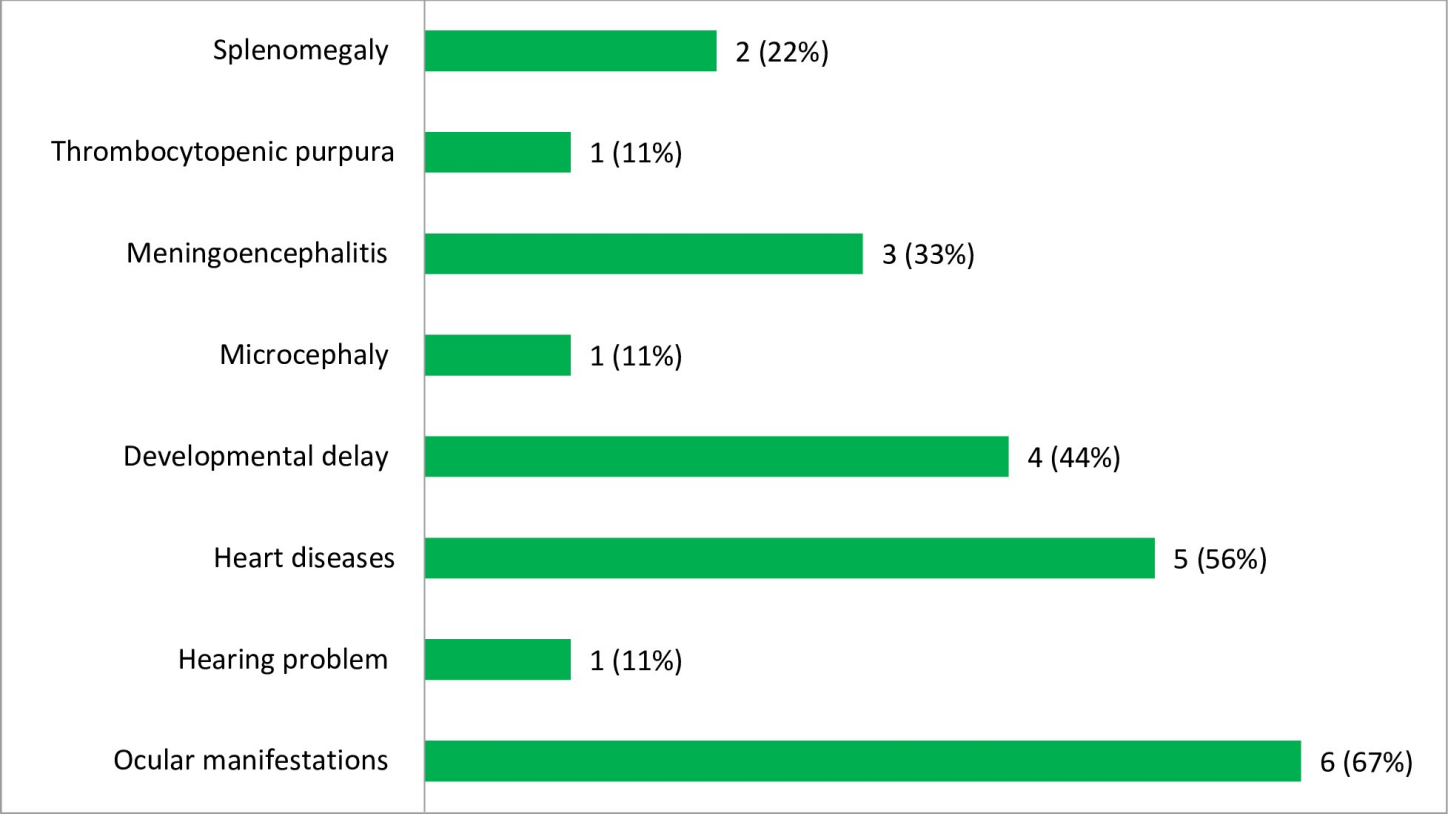
The frequency of different clinical manifestations among infants with laboratory confirmed CRS cases in Amhara Regional State Referral Hospitals, Northwest Ethiopia, December 2015-August 2017.

When we see the clinical manifestations of infants with the laboratory confirmed CRS cases, 6/9 (67%) and 5/9 (56%) of them had ocular manifestations and heart diseases, respectively. In addition, 1/9 (11%) of them had also hearing problems. Among the minor signs of the CRS cases, 4/9 (44%) and 3/9 (33%) of them had developmental delay and meningoencephalitis, respectively. Furthermore, 2/9 (22%) of them had also splenomegaly. Each of the microcephaly and thrombocytopenic purpura was accounted 1/9 (11%) in infants with laboratory confirmed CRS cases (Fig 4).

**Fig 5:**
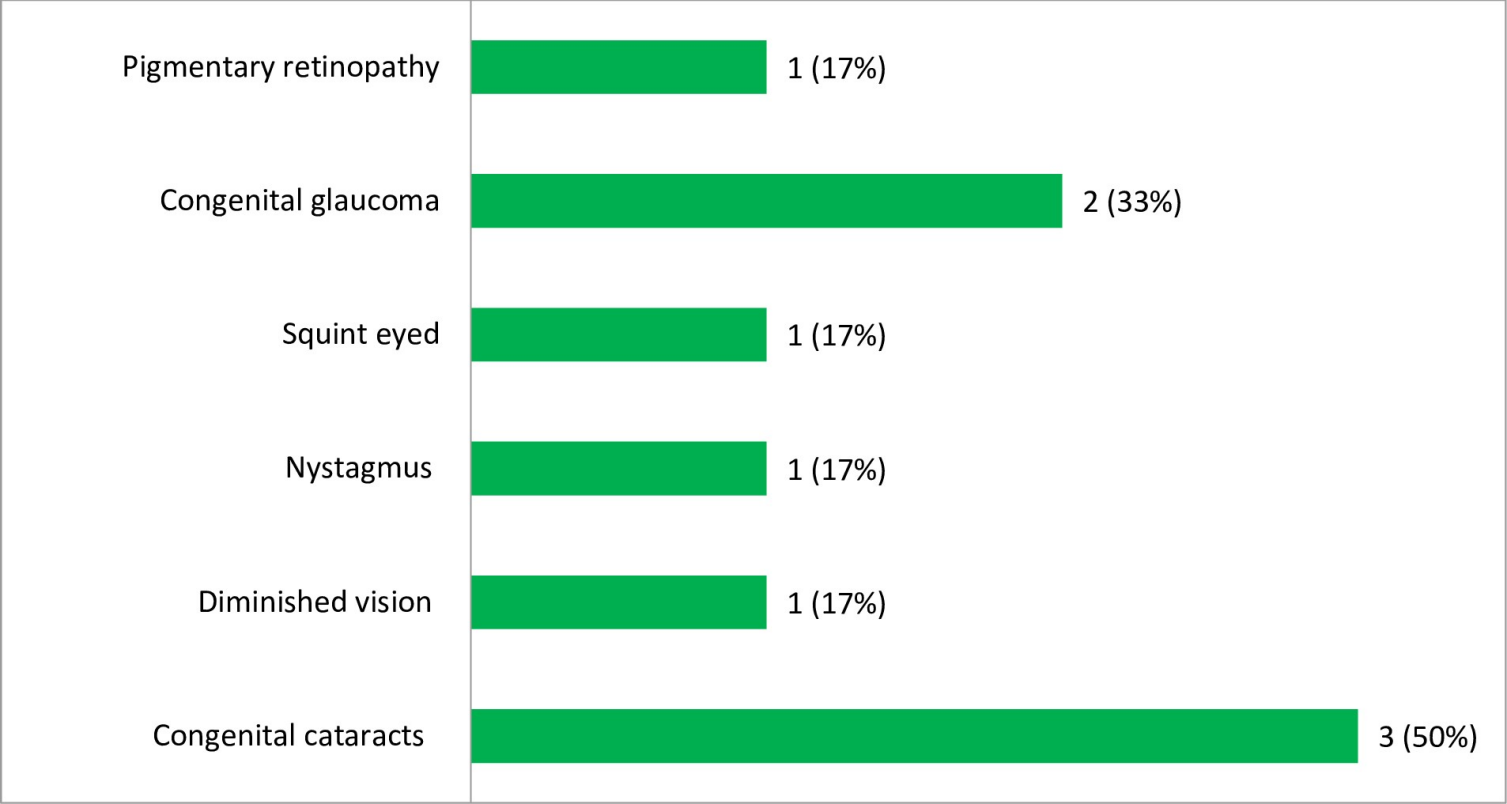
The frequency of different laboratory confirmed ocular manifestations among infants in Amhara Regional State Referral Hospitals, Northwest Ethiopia, December 2015-August 2017.

Of the total infants with laboratory confirmed ocular manifestations, 3/6 (50%) and 2/6 (33%) of the cases were congenital cataract and glaucoma, respectively. In addition, each of the pendular moment of the eyes (nystagmus), squint eyed, diminished vision and pigmentary retinopathy accounts 1/6 (17%) of the ocular manifestations (Fig 5).

According to the present study, 5/9 (56%) of the infants who had laboratory confirmed CRS were male and 6/9 (67%) of them were from urban settings. Of the total infants who had laboratory confirmed congenital rubella manifestations, two of them had only single major CRS manifestation (case 35 and case 50). However, the other seven infants with laboratory confirmed CRS had multiple clinical manifestations at a time and most of these multiple clinical presentations were among infants in the age groups of ≥ 1 months. The most common multiple symptoms in a given infant were congenital cataract, diminished vision, nystagmus, congenital heart disease, spleenomegaly, developmental delay and meningoencephalitis (case 17). However, all of the potential CRS cases had single clinical manifestations (case 6, case 13, case 36 and case 49) (Table 3).

**Table 3:** The overall clinical presentations of each infant suspected for CRS in Amhara Regional State Referral Hospitals, Northwest Ethiopia, December 2015-August 2017.

Cases	Age	Sex	Setting	Clinical presentation (s) in each infant
Case1	3 Months	Male	Urban	Congenital glaucoma
Case2	2 Months	Female	Urban	Heart disease
Case3	5 Months	Male	Rural	Cataract, nystagmus, congenital glaucoma
Case4	5 Months	Female	Urban	Heart disease, developmental delay *
Case5	4 Months	Male	Rural	Heart diseases, developmental delay
Case6	11Months	Female	Urban	Heart disease **
Case7	5 Months	Male	Urban	Heart disease, developmental delay
Case8	1 Day	Female	Rural	Congenital glaucoma, meningoencephalitis
Case9	9 Months	Male	Urban	Heart disease
Case10	11 Months	Female	Urban	Heart disease
Case11	1 Month	Male	Urban	Heart disease, meningoencephalitis
Case12	11 Months	Female	Urban	Heart diseases, thrombocytopenic purpura
Case13	8 Months	Female	Rural	Heart disease **
Case14	9 Months	Male	Rural	Heart disease
Case15	3 Months	Male	Urban	Squint eyed, developmental delay
Case16	11 Months	Male	Rural	Nystagmus, squint eyed
Case17	5 Months	Male	Urban	Cataract, diminished vision, nystagmus, heart disease, splenomegaly, developmental delay, meningoencephalitis*
Case18	7 Days	Female	Urban	Congenital glaucoma
Case19	9 Months	Male	Urban	Heart disease
Case20	11 Days	Female	Urban	Heart disease
Case21	11 Months	Female	Urban	Heart disease
Case22	4 Days	Male	Urban	Heart diseases, jaundice within 24 hours
Case23	1 Month	Female	Rural	Cataract
Case24	4 Months	Female	Rural	Cataract, hearing impairment*
Case25	2Days	Male	Urban	Cataract, meningoencephalitis, jaundice within 24 hours
Case26	5 Months	Female	Rural	Cataract
Case27	1 Day	Male	Urban	Congenital glaucoma
Case28	7 Days	Male	Urban	Splenomegaly, radiolucent bone disease
Case29	8 Days	Male	Urban	Cataract, jaundice within 24 hours
Case30	1 Day	Female	Rural	Heart disease, microcephaly*
Case31	2 Days	Male	Urban	Congenital glaucoma
Case32	4 Months	Male	Urban	Cataract, congenital glaucoma
Case33	1 Month	Female	Urban	Heart disease
Case34	2 Days	Female	Urban	Heart disease
Case35	1 Month	Male	Urban	Cataract*
Case36	11 Months	Female	Rural	Cataract **
Case37	7 Days	Male	Urban	Cataract
Case38	8 Days	Male	Rural	Pigmentary retinopathy
Case39	11 Months	Male	Rural	Heart disease, splenomegaly, developmental delay, meningoencephalitis*
Case40	10 Months	Male	Urban	Diminished vision, hearing impairment, splenomegaly, developmental delay
Case41	11 Months	Male	Urban	Squint eyed, glaucoma, heart disease, purpura, developmental delay, meningoencephalitis*
Case42	1 Month	Female	Urban	Heart disease, meningoencephalitis
Case43	6 Days	Female	Urban	Retinopathy, jaundice within 24 hours
Case44	1 Month	Male	Rural	Heart diseases
Case45	1 Month	Male	Urban	Retinopathy, thrombocytopenic purpura *
Case46	8 Days	Male	Urban	Cataract, nystagmus
Case47	11 Months	Female	Urban	Meningoencephalitis, diminished vision, blindness
Case48	3 Days	Male	Urban	Thrombocytopenic purpura, jaundice within 24 hours
Case49	11 Months	Male	Urban	Hearing impairment **
Case50	1 Month	Female	Urban	Congenital glaucoma*

Key

*: Laboratory confirmed CRS cases

**: Potential CRS cases, Cases without any star: Discarded CRS cases

Even though all the infants suspected for CRS were not born in the respective referral hospitals and difficult to get all live births in the target population in the catchment area, we have estimated the incidence of CRS by dividing the number of laboratory-confirmed CRS cases with the number of live births in the respective referral hospitals during the study period and multiplied by 1000. Since all of the infants with laboratory confirmed CRS cases were born in the respective referral hospitals and a total of 21846 live births were reported, the calculated incidence of the CRS cases in the study area was estimated to be 0.4 per 1000 live births.

## Discussion

Congenital rubella syndrome might present with a diverse form of clinical patterns which increases childhood morbidity and mortality [16, 33]. In the present study, 50 infants with different congenital malformations were suspected for CRS. Of these, 9/50 (18%) of them had rubella specific IgM positive results. According to the cases classified to the WHO case definitions, these might be categorized as a laboratory confirmed CRS cases. According to the present study, 4/14 (29%) of the infants ≥ 6 months of age had IgG positive results. As the maternal origin IgG is not mostly expected in this age category [34] and post natal rubella infection in less than one year infants is mostly uncommon [35], these might be the potential CRS cases.

Although WHO recommends rubella IgM as a laboratory conformation of CRS cases in most cases, serum IgM estimation alone might under-diagnose CRS cases when it compared to the combination of both the tests (IgM and IgG) in infants ≥ 6 months of age [36]. Rubella specific IgG test might be also more practical to diagnose CRS cases in infants ≥ 6 months [17, 37] using convalescent serum samples four weeks apart to see it’s persistent and to be used as confirmatory for CRS cases. But due to the nature of our study (cross sectional), we were unable to get the second sample to see the persistent of the IgG levels. Hence, those CRS cases with rubella IgG positive results with a single serum sample were classified as the potential CRS cases.

However, in despite of all these, the majority of the CRS cases, 37/50 (74%), in the present study were negative for either rubella specific IgM or IgG (for infants ≥ 6 months of age) antibodies. According to the cases classified to the WHO case definitions, these cases might be classified as non-CRS or discarded cases. This might be explained that the anomalies suspected for CRS might in the present study might be caused by other factors like genetic or environmental factors. They might be also caused by other infectious agents such as cytomegalovirus, *Toxoplasma gondii* and *Herpes simplex* [38, 39]. Furthermore, in the present day, other infectious agents like Zika virus can also cause congenital anomalies and severe birth defects [40]. Therefore, to help the patient, it might be also important to consider other teratogenic infectious agents or factors that can cause similar congenital anomalies like rubella.

The CRS cases might have different clinical presentations. In the present study, the major of the laboratory confirmed clinical anomalies were ocular manifestations (67%), heart related problems (56%) and hearing impairments (11%). Among the ocular manifestations, 50% of the cases were congenital cataract followed by congenital glaucoma (33%). This can be explained that in embryo, the most rapid development of heart muscle occurs along with the development of the inner ear and lens. Therefore, damage caused by congenital rubella infection in the ears and eyes of the fetus might be often accompanied by a variety of heart defects [41].

The pattern of the clinical presentations of the CRS cases in the present study was also consistent with studies in Oman and Madurai, India [42, 43]. However, in another study in Bangladesh, the most frequently observed congenital rubella syndrome manifestation was neurological problems followed by ocular problems and congenital hearing loss [16]. This difference in the clinical presentation of the CRS cases in different studies might be associated with the specialty of the health institutions in which those studies carried out. Even though it is difficult to speculate properly, the difference might be also related to the organ system involved and the gestational age at which the maternal rubella infections occurred [44]. The eye and heart defects often follow infection during the first 8 weeks of pregnancy, whereas brain damage and deafness are likely to be seen when maternal infection occurs in the first 18 weeks of pregnancy [45]. All these indicate that most CRS cases occur when women acquire rubella infection during early pregnancies [46, 47].

In the present study, of the total laboratory confirmed CRS cases, 5(56%) of them were males. Since the reason is not clear and the number of cases is small, it is difficult to conclude that CRS is more common male infants. Therefore, further large scale study in nation wise is needed. However, a similar findings were also reported in other studies in Italy [48], Bangladesh (16) and Pakistan [49]. In this study, the majority of the infants with CRS cases (67%) were from urban settings. This can be explained that in the urban environment, there might be high population density as compared to the rural areas. This overcrowded living condition in a given area can contribute for the air born transmission of rubella virus in susceptible groups like child bearing women that indirectly contribute the development of CRS cases. However, since our study has been done only in three randomly selected health facilities and include only infants seeking to get health care and visit the respective hospitals, this might not show the actual burden of CRS in the region as well as in the country. Therefore, community based, multicenter and large scale study in different regions of the country might be required to see the real difference of CRS in urban and rural setting. In addition, there might be also differences between the urban and rural populations in seeking the health facilities and CRS cases would be remained undiagnosed rural settings. Hence, strong CRS surveillance system might be crucial to see the burden of the CRS cases in both urban and rural settings.

According to the present study, the majority of the laboratory confirmed CRS cases (19%) were among infants less than 6 months. Only 14% of infants ≥ 6 months have IgM positive results. This can be explained that, despite the rubella specific IgM antibody usually starts to declines after 2 months of age [50], most of the congenitally infected infants might have also detectable rubella IgM positive results up to 6 months of life [51]. However, only one-third CRS cases might have detectable IgM from 6 months up to one year, and occasionally it might even persists for up to two years of life [51, 52]. These indicate that the rubella specific IgM might be almost positive in the first 6 months of age if the suspected congenital anomalies are realty due to the teratogenic congenital rubella infections. However, the IgM positivity might be decreases as the ages of the infants increased.

In the present study, the clinical presentations of the laboratory confirmed CRS cases varied in the different age categories. Some of the infants with laboratory confirmed CRS cases had only single clinical manifestation but some others had multiple clinical symptoms at a time. This variation in the clinical presentations can be explained that a child might have multiple birth defects and some of these birth defects would be recognized immediately after birth and some others might be revealed after months or years [53]. Furthermore, as many as 50% of infants with CRS may appear normal at birth [54], but they will have late-onsets of congenital rubella manifestations. In mild forms of CRS, there might not be obvious clinical manifestations at birth and the onset of CRS-related symptoms can be delayed [55, 56]. However, an infant who have already acquired rubella infection congenitally might be IgM positive immediately after births even without congenital rubella manifestation. This can be categorized as congenital rubella infection (CRI) only. But in some cases, they might have late-onset rubella associated congenital anomalies as discussed earlier or they might not develop any rubella malformations at all.

As the birth registration coverage is very minimal in Ethiopia [57], it is very difficult to get the total number of live births from the target population of the catchment areas of the study sites during the study period. Hence, despite with its limitation, we have calculated the incidence of CRS by considering the number of live births in the respective referral hospitals. Accordingly, the incidence of CRS was 0.4 per 1000 live births. This result is in line with the global incidence of the CRS in the pre-vaccine era in 1969 [58]. Prior to the introduction of rubella vaccine, the incidence of CRS varied from 0.1–0.2/1000 live births during endemic periods and 0.8-4/1000 live births during rubella epidemics [59, 60].

The CRS incidence in our study was also comparable with other previous surveillance reports like 0.7 per 1000 live births in Oman [61] and 0.5 in Malaysia [62] before they introduced rubella vaccine in their immunization program. A similar incidence of CRS (0.77 per 1000 live births) was also estimated in Indonesia using transmission modelling [63]. But, the incidence of the CRS cases in our study was lower than other previous reports in the pre-vaccine era like 2.2 per 1,000 live births in Panama, 1.7 in Israel, 1.7 in Jamaica, and 1.5 in Singapore during rubella outbreaks [59]. However, the incidence of the CRS in the present study was higher than 0.1 per 100,000 live births in Australia, which introduced rubella vaccine in its national immunization programme [64]. These CRS incidence variations in different populations in different countries might be due to the fact that there might be differences on the number of susceptible pregnant women, the circulation of rubella virus, the presence or absence of rubella containing vaccine in their national immunization program and differences in the vaccination coverage in countries in which rubella vaccination has already included in their national immunization programmes.

## Conclusion

In this study, many infants had laboratory confirmed CRS cases and most of them had multiple rubella associated congenital defects at a time. According to the present study, the most commonly clinical presentation of the CRS cases was ocular manifestation followed by congenital heart diseases. The incidence of the CRS detected in the present study was similar to the global incidence of the CRS in the pre-vaccine era. Based on our finding, we would like to recommend strong CRS surveillance system in the study area as well as in the country. This might help to show the burden of CRS for decision makers and to see the progress in the near feature. We would like also recommend the introduction of rubella containing vaccine in the national immunization program to reduce the rubella/CRS associated illness in the country.
